# Quantitative Electroencephalography Monitoring in Type A Aortic Dissection Surgery: A Clinical Case Review and Prospective Applications

**DOI:** 10.1002/brb3.70086

**Published:** 2024-10-08

**Authors:** Ya‐Peng Wang, Jason Z. Qu, Dong‐Jin Wang

**Affiliations:** ^1^ Department of Cardiothoracic Surgery Nanjing Drum Tower Hospital, Chinese Academy of Medical Sciences & Peking Union Medical College Nanjing Jiangsu China; ^2^ Department of Anesthesia, Critical Care and Pain Medicine Massachusetts General Hospital, Harvard Medical School Boston Massachusetts USA; ^3^ Department of Cardiothoracic Surgery Nanjing Drum Tower Hospital, The Affiliated Hospital of Nanjing University Medical School Nanjing Jiangsu China

**Keywords:** aortic dissection, brain function, quantitative electroencephalography

## Abstract

**Purpose:**

This review explores advanced methods for assessing perioperative cerebral function in Type A aortic dissection (TAAD) patients, with a focus on quantitative electroencephalography (QEEG). It highlights the critical issue of cerebral malperfusion, which is associated with higher mortality and poor prognosis during the perioperative phase in TAAD patients.

**Method:**

The review centers on the utilization of QEEG as a pivotal tool for the extensive monitoring of brain function at various stages: preoperatively, intraoperatively, and postoperatively. It elaborates on the foundational principles of QEEG, including the mathematical and computational analysis of electroencephalographic signals, enriched with intuitive graphical representations of cerebral functional states.

**Finding:**

QEEG is presented as an innovative approach for the real‐time, noninvasive, and reliable assessment of cerebral function. The review details the application of QEEG in monitoring conditions such as preoperative cerebral malperfusion, intraoperative deep hypothermic circulatory arrest, and postoperative recovery of cerebral function in patients undergoing TAAD treatment.

**Conclusion:**

Although QEEG is still in an exploratory phase for TAAD patients, it has shown efficacy in other domains, suggesting its potential in multimodal brain function monitoring. However, its broader application requires further research and technological advancements.

## Graphical abstract

The crux of perioperative monitoring of TAAD lies in the continuous assessment of central nervous system function, which can be achieved through a multimodal approach encompassing NIRS for monitoring oxygen delivery, QEEG for evaluating neurological function, and TCD for assessing blood flow, with QEEG potentially aiding in the early detection of neurological abnormalities, though further research and development is warranted in this regard.

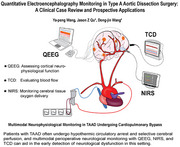

## 1 Introduction

Type A aortic dissection (TAAD) is a life‐threatening condition that requires prompt surgical intervention. Despite improved treatment technology, the mortality rate in TAAD patients remains high due to neurologic dysfunction from cerebral malperfusion (Members et al. 2022). Swift restore of cerebral perfusion is imperative for the survival of patients with TAAD. Emergency surgical repair is the standard care for patients with TAAD, and cerebral protection is the major component of surgical planning (Tanaka et al. 2005). For most surgeons, deep hypothermic circulatory arrest (HCA) is still the preferred surgical technique for TAAD repair to avoid or minimize postoperative neurologic complications. Although prolonged durations of cardiopulmonary bypass (CPB) and embolization of thrombi from the aortic false lumen are among many risk factors for postoperative brain injury, the mechanism of postoperative neurologic dysfunction is largely unknown (Qu et al. 2021). The incidence of neurologic injury during the perioperative period of TAAD has been reported to be as high as 30% (Krüger et al. 2011). Despite the improvement of surgical technology, including intraoperative hemodynamic monitoring such as transesophageal echocardiography and supplemental cerebral perfusion during circulatory arrest, there are limited modalities of cerebral monitoring (Qu et al. 2021). There are currently three commonly used noninvasive cerebral monitoring technologies: electroencephalography (EEG) or processed EEG, transcranial Doppler (TCD) and near‐infrared spectroscopy (NIRS). With own limitations, each has shown its value and limitation in predicting neurologic outcomes. TCD can only measure blood flow in large intracranial vessels with very limited windows to obtain good quality images (Qu et al. 2021). NIRS primary measures regional cerebral oxygen from the anterior and middle cerebral arteries (Zheng et al. 2013). Lewis et al. (2018) and Urbanski et al. (2013) Furthermore, the correlations between the above monitoring and neurologic outcomes have not been validated in large randomized trials.

EEG provides insightful data on the brain's electrical activity and holds potential for refining patient prognosis (Keenan et al. 2016). However, the interpretation of raw EEG data is complex and can be difficult to apply in a clinical setting. To address this challenge, the use of quantitative EEG (QEEG) monitoring has been proposed for the assessment of perioperative cerebral function in TAAD patients (Wang, Lu, et al. 2023; Wang, Liu, et al. 2023). We introduce the application of QEEG monitoring in the perioperative period in patients with TAAD.

## 2 QEEG Monitoring

**FIGURE 1:**
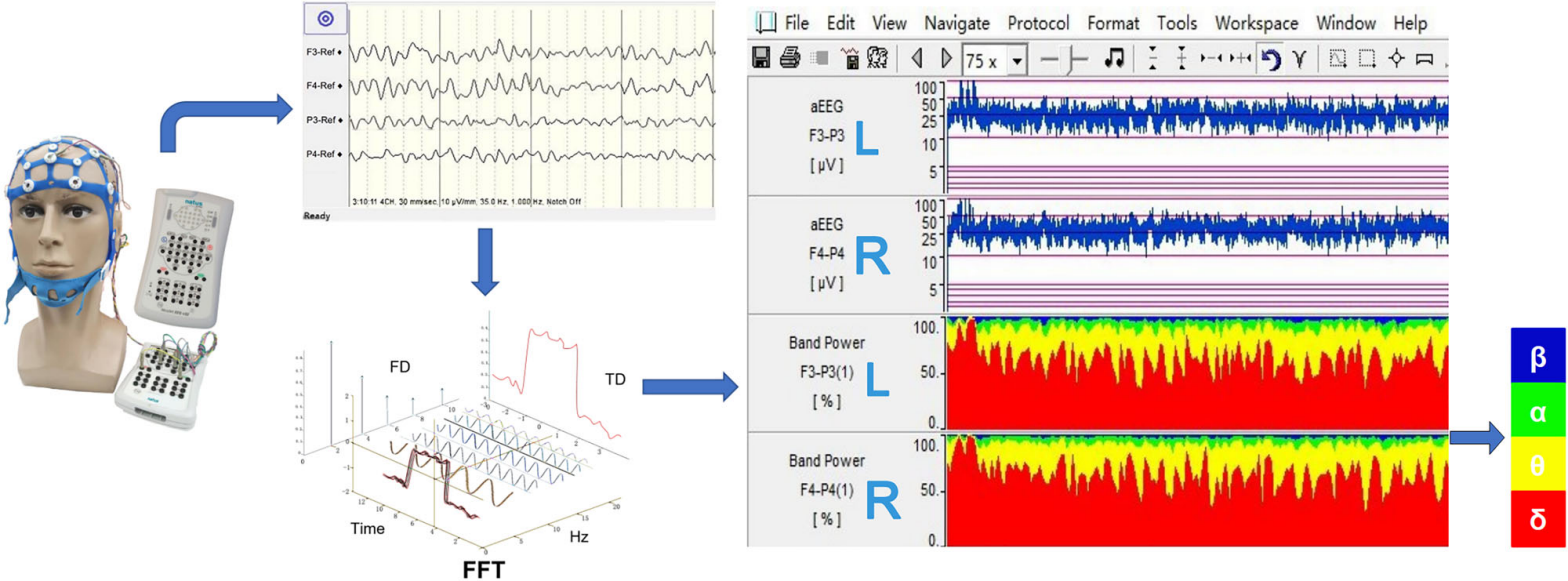
Schematic diagram of raw EEG signal processing and conversion to QEEG. EEG, electroencephalography; FD, frequency domain; FFT, fast Fourier transform; L, left hemisphere; QEEG, quantitative electroencephalography; R, right hemisphere; TD, time domain.

QEEG utilized mathematical and computational strategies to analyze electroencephalographic signals. This method condensed voluminous EEG data into a concise graphical representation, offering a visual and numerical illustration of a patient's cerebral activity. The employment of QEEG for monitoring facilitated swift assessment and interpretation of cerebral function by clinicians without specialized training in neuroelectrophysiology, directly at the patient's bedside. The QEEG monitoring system encompassed various elements (Figure 1 ), such as data acquisition devices, computing hardware, analytical software, and signal amplifiers, integrating these components to provide a comprehensive evaluation of brain function (Jordan 1999; Scheuer and Wilson 2004).

**FIGURE 2:**
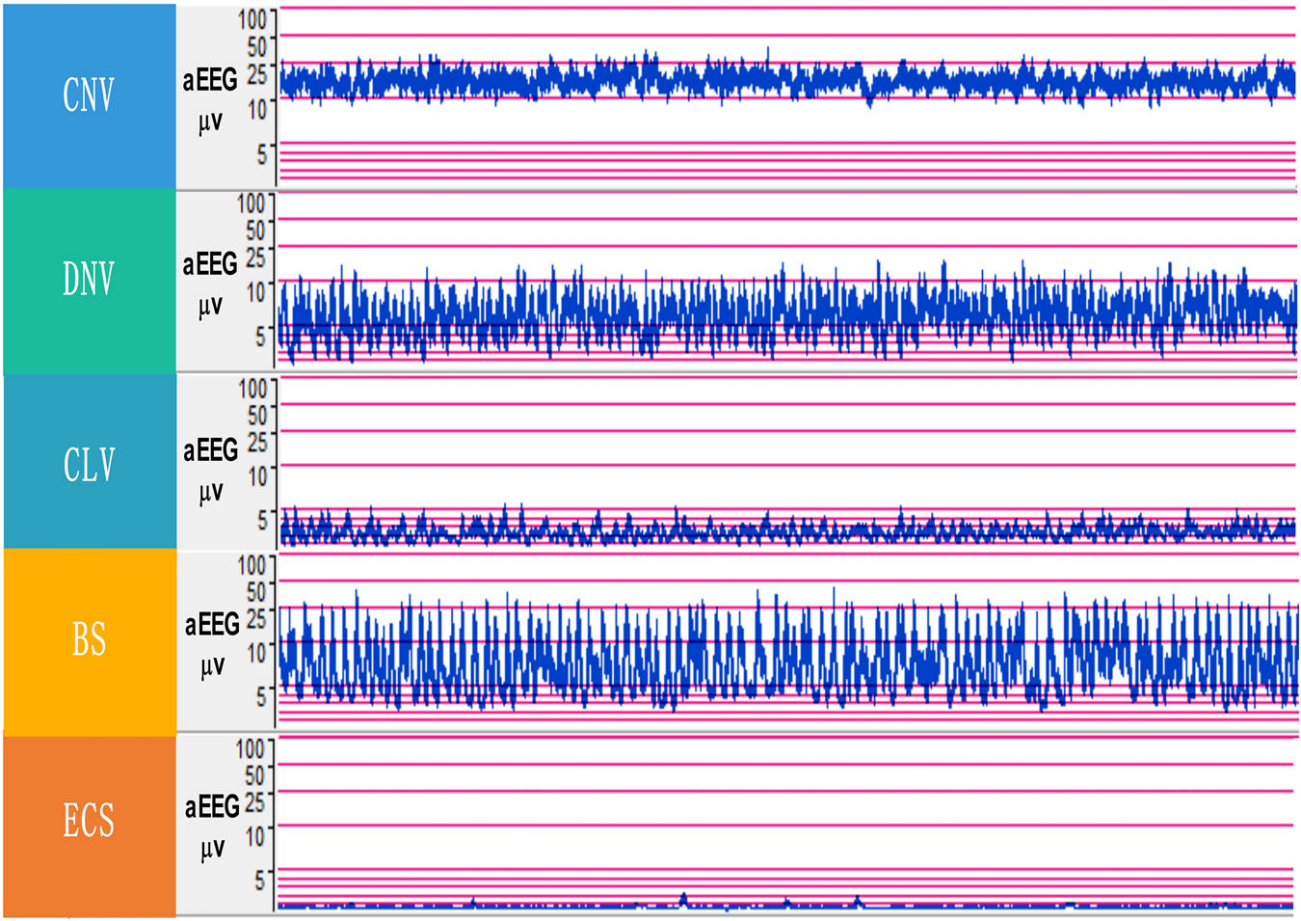
Background activities of amplitude‐integrated EEG. aEEG, amplitude‐integrated electroencephalography; BS, burst suppression; CLV, continuous low voltage; CNV, continuous normal voltage; DNV, discontinuous normal voltage; ECS, electrocerebral silence; EEG, electroencephalography.

### 2.1 Background Activities of Amplitude‐Integrated EEG (aEEG) (Figure 2)

#### 2.1.1 Continuous Normal Voltage (CNV)

The upper threshold of aEEG usually falls between 10 and 50 µV, whereas the lower threshold commonly spans from 5 to 10 µV. With the signal's bandwidth deemed normal, the outlook for individuals presenting with CNV is typically regarded as favorable.

#### 2.1.2 Discontinuous Normal Voltage (DNV)

DNV is identified in EEG signals by its intermittent activity and fluctuating voltage limits. The signal's upper limit commonly exceeds 10 µV, whereas its lower limit is variable, usually not surpassing 5 µV. This signal often exhibits a bandwidth broader than the norm, accompanied by mild irregularities in aEEG readings. Despite such deviations, the prognosis for individuals demonstrating DNV is generally viewed as positive.

#### 2.1.3 Continuous Low Voltage (CLV)

CLV denotes an EEG signal pattern marked by ongoing low‐amplitude activity, where the upper voltage limit does not exceed 5 µV. Compared to standard EEG signals, its bandwidth is typically reduced. The occurrence of CLV suggests significant damage to the cerebral cortex and is frequently linked with a grim prognosis, barring the impact of substantial doses of sedative medications.

#### 2.1.4 Burst Suppression (BS)

BS is identified as an EEG pattern featuring cycles of high‐amplitude bursts followed by phases of low‐amplitude or no activity. This pattern is characterized by an upper voltage limit exceeding 25 µV and a lower limit of 10 µV or less, with a bandwidth broader than that seen in typical EEG signals. The occurrence of BS marks a profound deviation in EEG readings, frequently indicative of severe ischemia, hypoxia, or profound sedation. The prognosis associated with the presence of BS is generally adverse, notwithstanding the effects of sedative medication.

#### 2.1.5 Electrocerebral Silence (ECS)

ECS is distinguished by a predominant lack of electrical activity in the background EEG pattern. The amplitude's upper limit falls below 5 µV, sometimes even reaching as low as 2 µV, accompanied by an exceedingly narrow bandwidth. This pattern suggests a significant dysfunction of the cerebral cortex or the occurrence of brain death, marked by the flat trace. Consequently, the prognosis in the presence of ECS is deemed very poor.

### 2.2 The Principle of Relative Band Power (RBP)

**FIGURE 3:**
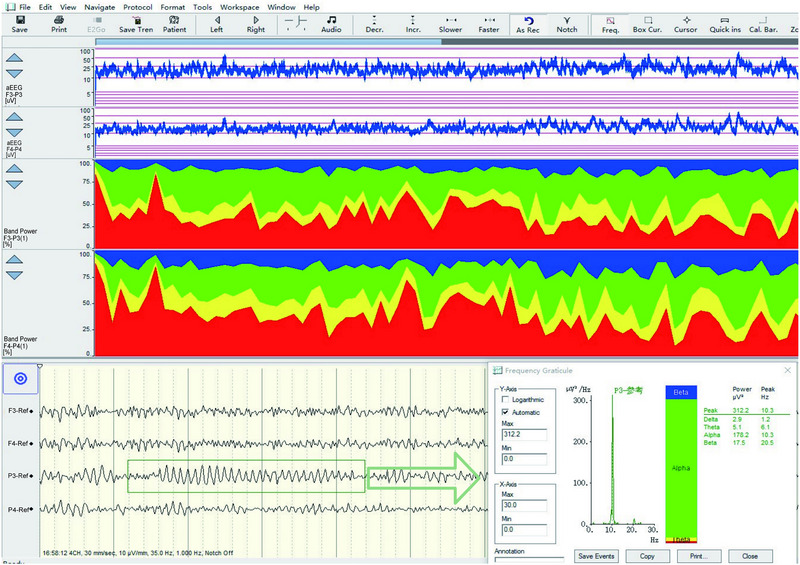
Trend chart of QEEG. QEEG, quantitative electroencephalography.

RBP serves as a crucial metric for observing variations in EEG frequency bands. It is determined by the proportion of energy within a specific frequency band relative to the total energy across all bands, expressed as a percentage. This metric is visually depicted through distinctive color zones in a graphical format (Figure 3). Within this representation, the blue area denotes the area under the curve for the β frequency band, green for the α band, yellow for the θ band, and red for the δ frequency band (Figure 3). RBP offers a valuable means for examining shifts in EEG frequency patterns, boasting potential utility in neuroscience and psychology domains.

**FIGURE 4:**
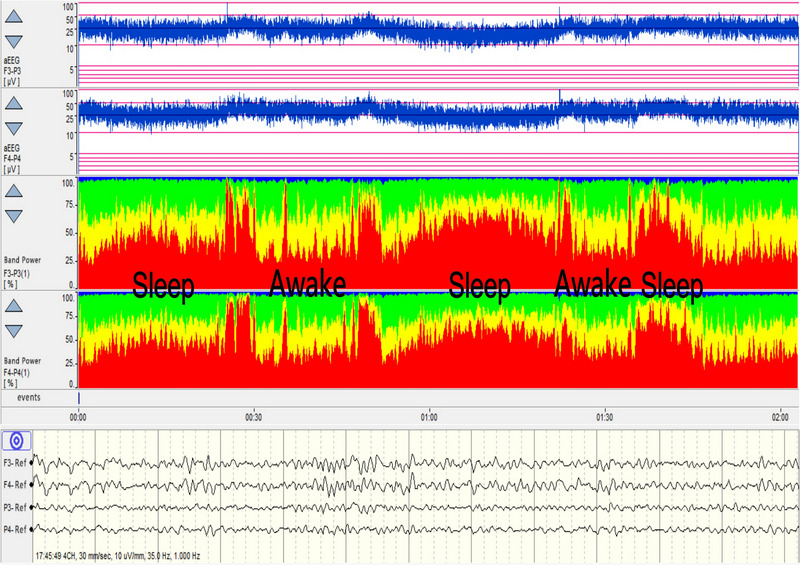
Sleep–wake cycle.

**FIGURE 5:**
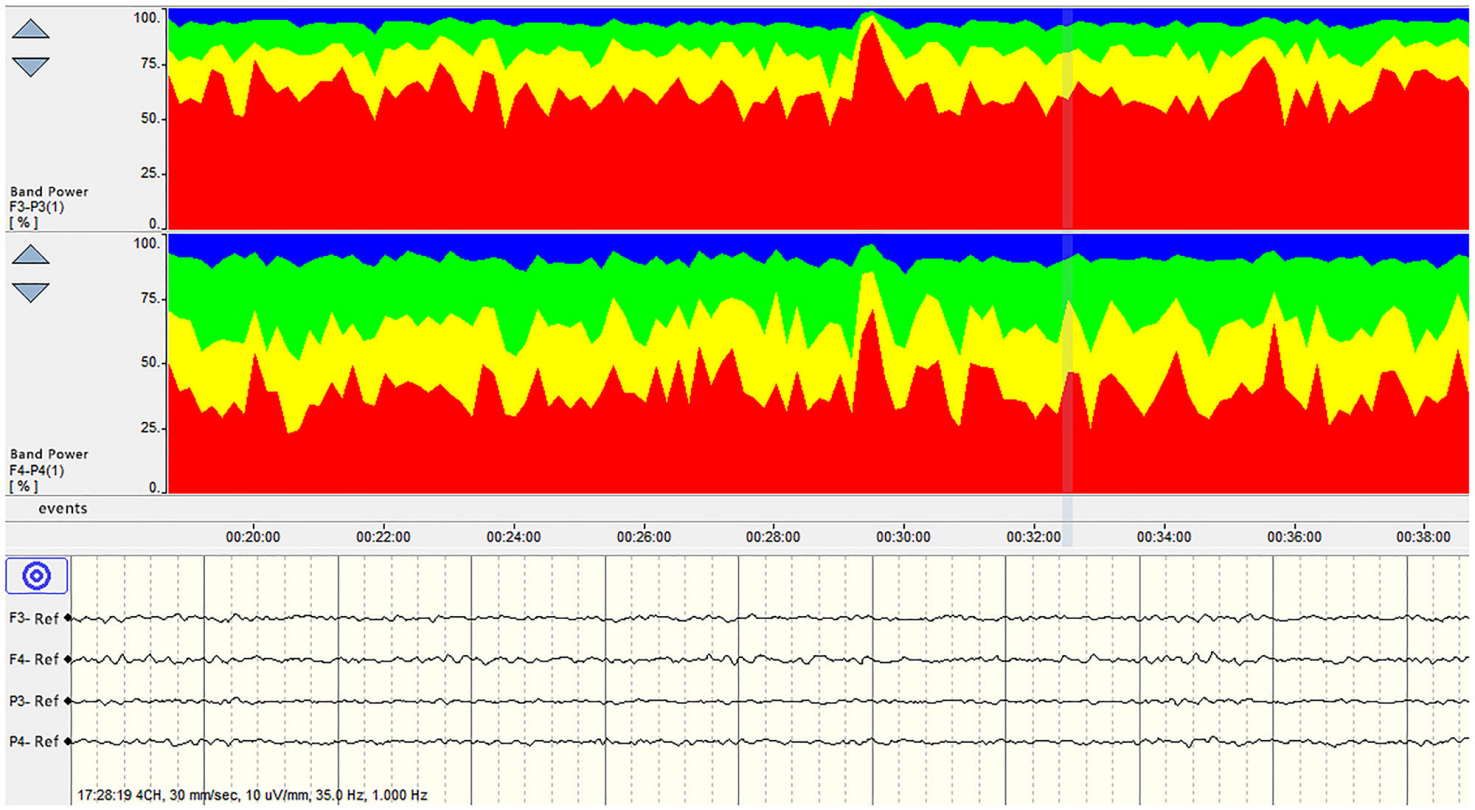
The RBP delta in the left hemisphere is higher than in the right hemisphere, indicating a potential left‐sided cerebral injury. RBP, relative band power.

In healthy adults, background EEG activity predominantly features α rhythms during states of wakefulness, calmness, and with eyes closed. Utilizing the principle of RBP for analysis, it is observed that α wave constitutes over 80% of EEG activity, with minimal contributions from δ, β, and θ waves. As sleep transitions from light to deep stages, an increase in δ and θ wave activities is noted alongside a reduction in α and β wave activities, culminating in the predominance of δ waves in deep sleep. This alteration in EEG trends offers insights into the integrity of an individual's sleep cycle (Figure 4). A robust sleep‐wake rhythm in normal adults is indicative of healthy cerebral cortex function. Additionally, EEG activities in corresponding areas of the two cerebral hemispheres exhibit synchrony and similarity, evidenced by agreement between aEEG and RBP data. Damage to one cerebral hemisphere leads to altered EEG activity in the affected region, disrupting the consistency between RBP or aEEG readings of that area and its counterpart in the unaffected hemisphere. Such asymmetry is clearly discernible in the RBP trend chart, facilitating the assessment of the cerebral hemispheres’ symmetry (Figure 5).

## 3 Application of QEEG in Patients with TAAD and Cerebral Malperfusion

Patients with TAAD have a high risk of neurologic injury that, if not detected and treated in time, may lead to permanent neurological dysfunction and death. QEEG provides information on cerebral cortical function and helps to recognize early brain injuries that prompt timely intervention.

### 3.1 Preoperative Concomitant Cerebral Malperfusion

#### 3.1.1 Preoperative Concomitant Transient Impairment of Consciousness

**FIGURE 6:**
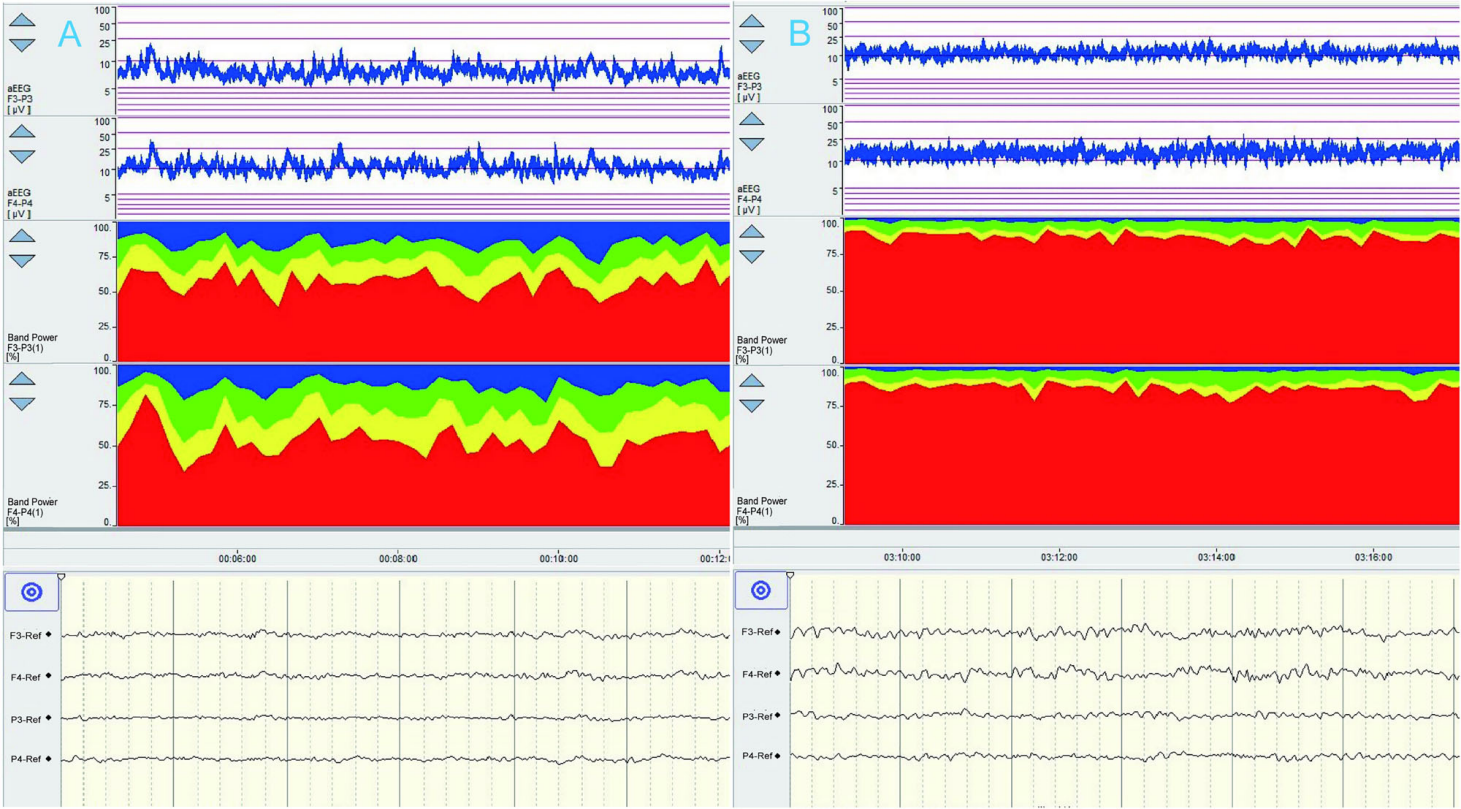
Preoperative and postoperative aEEG changes and the potential impact of anesthetic agents. Preoperative evaluation showed increased β waves and decreased α waves, suggesting stress. Notably, the left‐sided aEEG had reduced amplitude compared to the right (A). Two hours post‐surgery, aEEG showed symmetry between hemispheres, despite elevated theta waves, possibly linked to anesthetic effects on cerebral activity (B). F3‐P3 and F4‐P4 corresponded to the left and right hemispheres. aEEG, amplitude‐integrated electroencephalography.

Transient loss of consciousness (TLOC) prior to surgery in patients with TAAD is primarily attributed to transient ischemic attack (TIA) resulted from instability of cerebral blood supply. A study from IRAD included 2402 patients and showed 15% patients with TAAD presented with cerebral ischemia (Sultan et al. 2021). This was consistent with the incidence of neurologic dysfunction in a study from our hospital (Xue et al. 2021). In this study, 7.5% patients presented as TIA (Xue et al. 2021). The mechanism of TIA in patients with TAAD is largely unknown, whereas it is speculated that occlusion or compression of the head vessels by the intimal flap or enlarged false lumen of the head vessel is likely the cause. A significant increase in β waves and a reduction in α waves suggest brain under distress. The patient in Figure 6 presented with aortic dissection (back pain) for 8 h experiencing TLOC before slipping into a coma before emergency surgery.

Enhanced α waves and improved brain function following aortic arch repair suggested the cerebral blood flow has been restored. These outcomes implied that early surgical intervention brain injury in patients with TLOC.

#### 3.1.2 Preoperative Coma

Coma is an ominous sign of patients with TAAD and often considered a contraindication for surgical intervention. However, the severity of coma varies widely duration and depth of loss of consciousness and underlying cerebral‐vascular pathology. Although coma > 3 h was considered a contraindication for open aortic surgery (Tanaka et al. 2005), numerous cases have reported favorable neurological outcomes following TAAD repair in patients presented coma for > 12 h (Pocar et al. 2006). Neurological evaluation or monitoring tools that provide predictive values are lacking.

**FIGURE 7:**
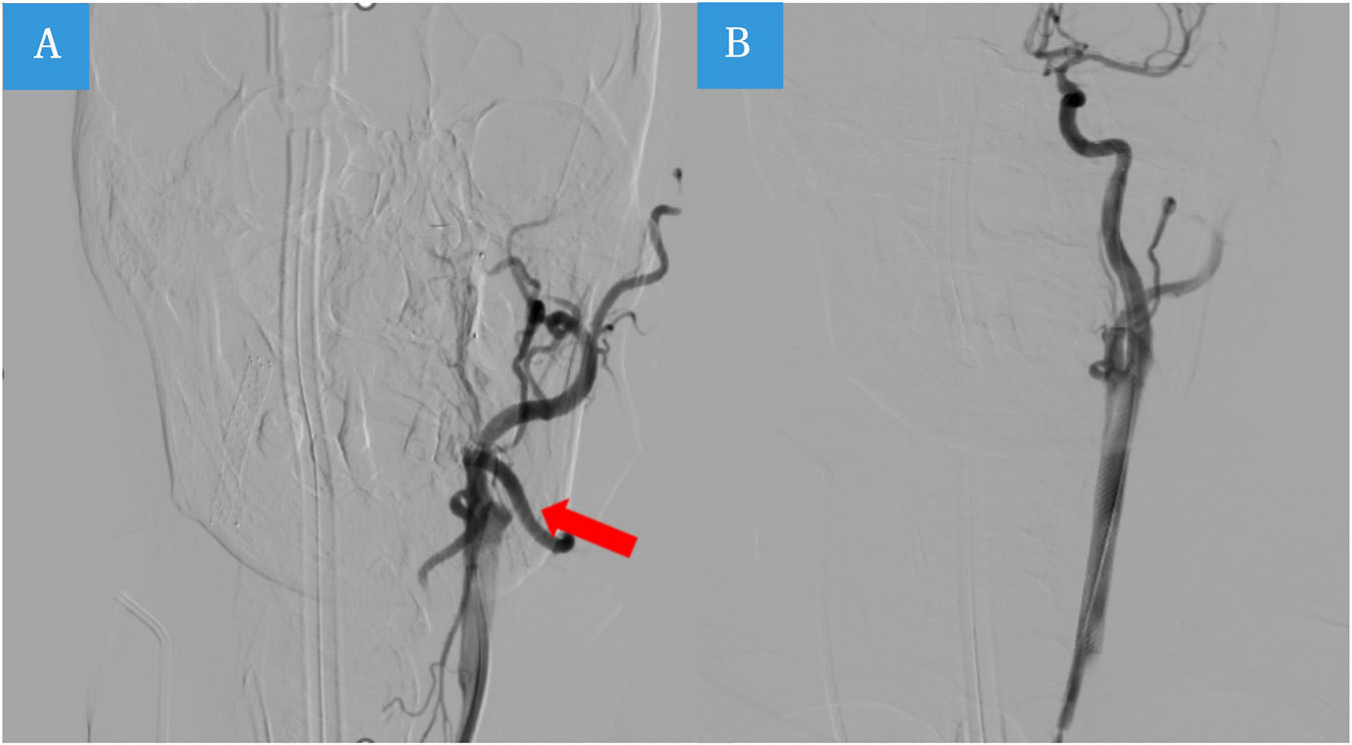
Vascular recanalization after stent placement in the left internal carotid artery. Red arrows occlusion of the LICA (A); Angiography after LICA Stenting (B).

**FIGURE 8:**
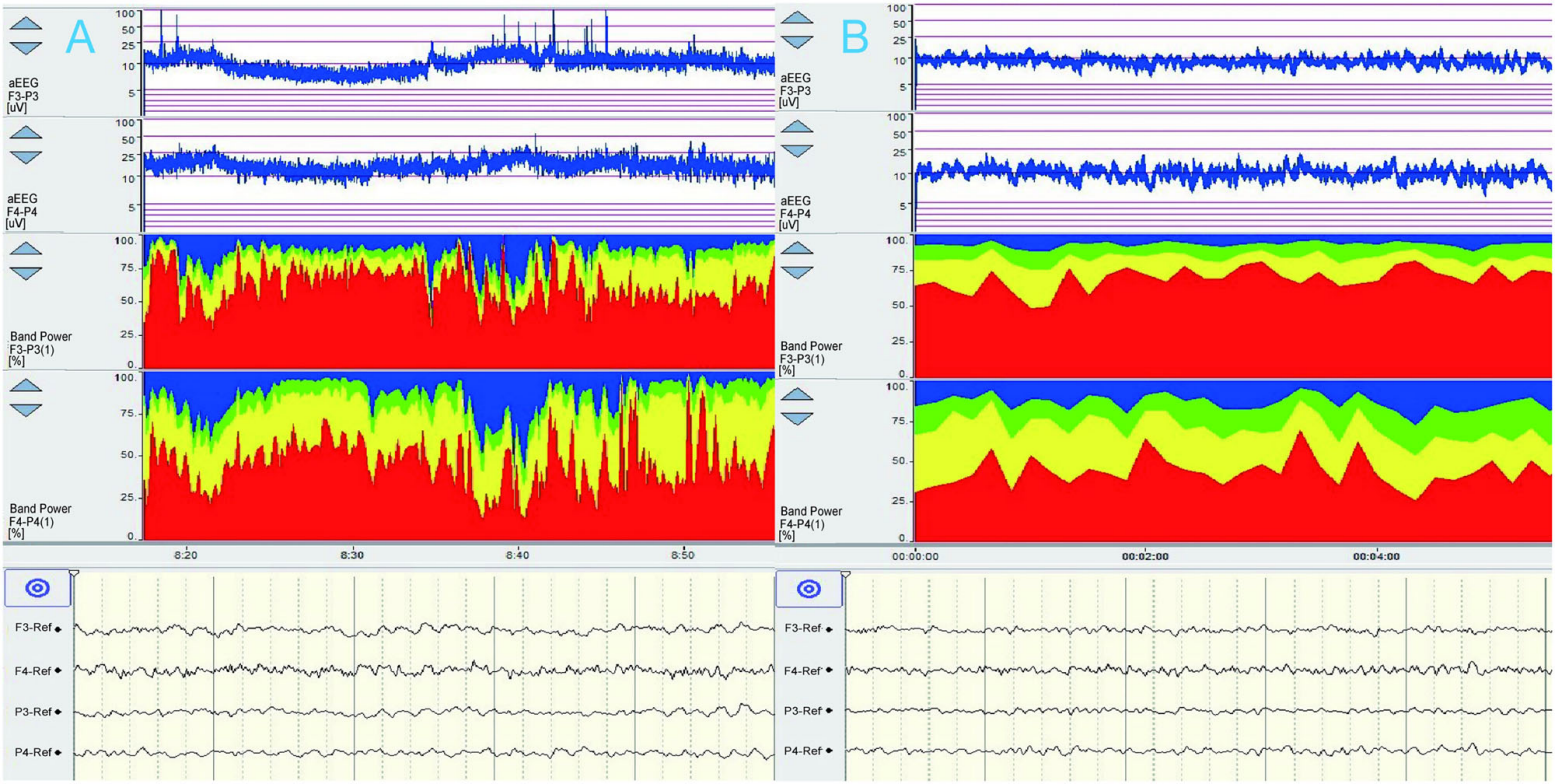
Pre‐ and post‐surgery QEEG brain activity. On the first day of admission, QEEG monitoring showed symmetrical aEEG in both hemispheres, but δ waves were significantly more prominent in the left hemisphere, indicating severe left brain injury (A). After interventional neuroradiology, QEEG revealed a gradual increase in α waves in the RBP (B). F3‐P3 and F4‐P4 corresponded to the left and right hemispheres, respectively. aEEG, amplitude‐integrated electroencephalography; EEG, electroencephalography; QEEG, quantitative electroencephalography; RBP, relative band power.

We previously reported a 53‐year‐old patient of TAAD presented with coma for 9 h (Wang et al. 2023). CT angiogram on admission showed occlusion of the LICA (Figure 7A). A QEEG showed dominant δ wave on the left hemisphere suggesting ischemia consistent with angiographic findings (Figure 8A); however, the overall aEEG remained symmetrical in both hemispheres. He underwent ascending aortic replacement with total aortic arch and descending thoracic aortic stenting. Postoperative QEEG revealed increase amplitude in α waves in the RBP (Figure 8B), which was consistent with revascularization surgery (Figure 7B). The patient was discharged home 52 days postoperatively. At his 2‐week follow‐up, he showed no cognitive dysfunction and normal motor function of left extremities albeit right‐sided hemiplegia.

#### 3.1.3 Preoperative Cardiac Arrest and Aortic Dissection Rupture

**FIGURE 9:**
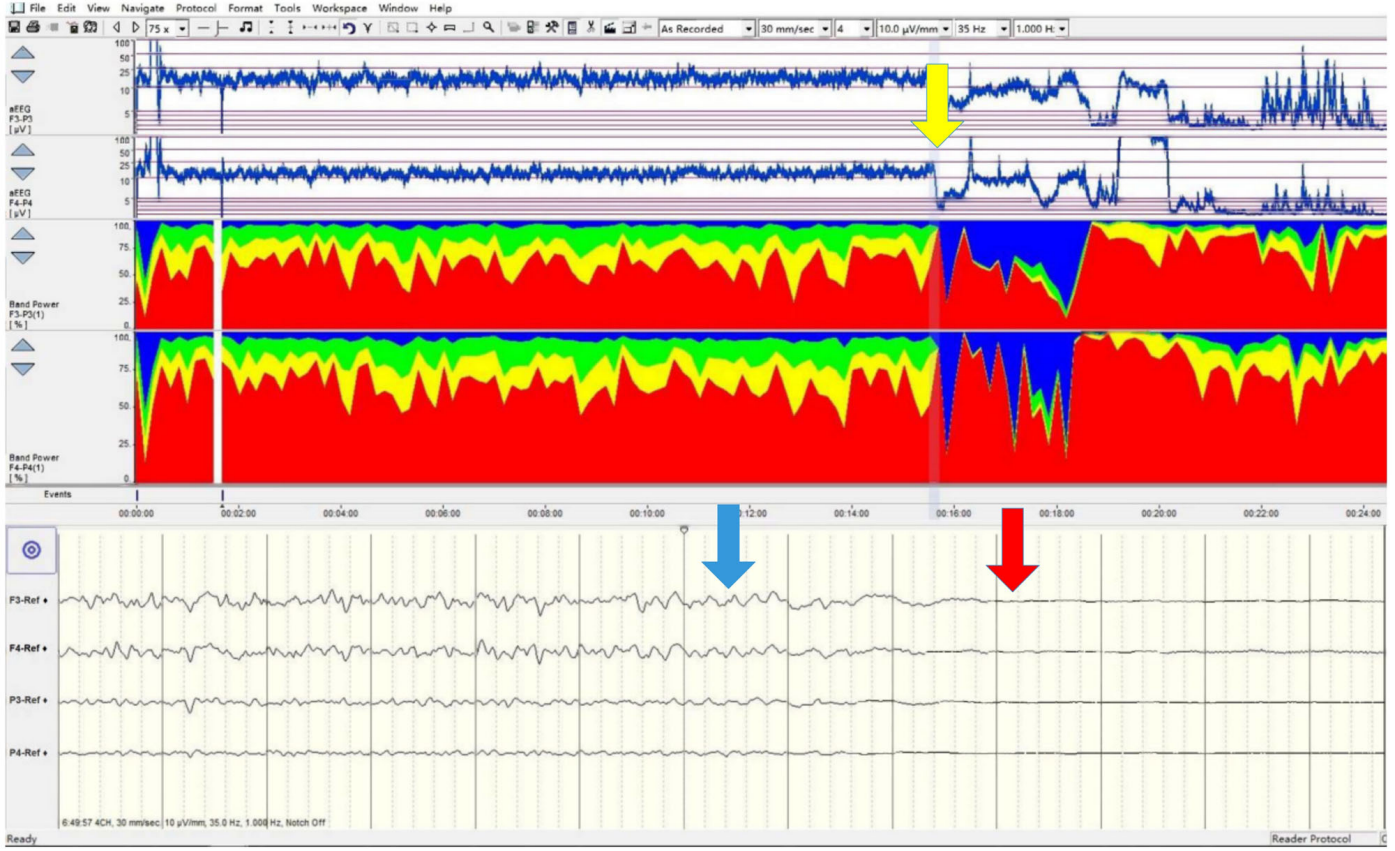
Electroencephalographic changes before, during, and after aortic dissection rupture. The blue arrow indicates baseline EEG before aortic dissection rupture, showing typical activity. The red arrow marks the rupture, characterized by a flattened waveform, suggesting significant brain function compromise. The yellow arrow shows EEG interference from chest compressions during CPR, highlighting the impact of external forces. F3‐P3 and F4‐P4 correspond to the left and right hemispheres. EEG, electroencephalography.

A 72‐year‐old man was admitted to our department after experiencing a syncopal episode lasting more than 9 h. Aortic CTA imaging confirmed TAAD with an occlusion of the right internal carotid artery. As the patient was going into cardiac arrest secondary to rupture of the aortic dissection, aEEG monitoring revealed noticeable interhemispheric differences, with the aEEG on the right side showing lower upper limit values than the left and a mild increase in the proportion of slow waves, and EEG waveform eventually flattened (Figure 9).

### 3.2 Intraoperative Changes in QEEG for TAAD Surgery

Surgical technology in aortic arch surgery requiring circulatory arrest has evolved significantly as the introduction of DHCA (Griepp et al. 1975). Much of the focus is on neuroprotection, that is, hypothermia and cerebral perfusion; however, there is a great demand for intraoperative cerebral monitoring (Qu et al. 2021).

As the temperature was lowered, the aEEG gradually decreased from its continuous baseline, eventually reaching a state of electrical silence. Following the completion of HCA, the process of rewarming and the reinstatement of extracorporeal circulation led to a gradual restoration of aEEG activity, eventually returning to its baseline level of continuity. QEEG could serve as a tool for assessing the effectiveness of hypothermic treatment.

**FIGURE 10:**
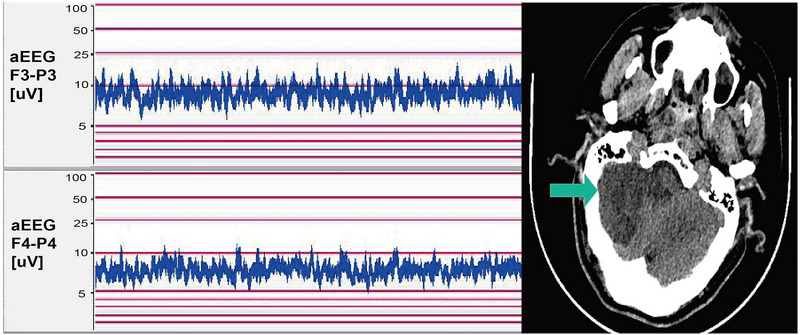
Post‐rewarming QEEG changes and corresponding brain CT findings. Following rewarming, QEEG initially showed symmetrical aEEG readings, but a progressive decrease in the right aEEG was observed. Postoperative brain CT later confirmed a large cerebral infarction in the right hemisphere. F3‐P3 and F4‐P4 corresponded to the left and right hemispheres. aEEG, amplitude‐integrated electroencephalography; QEEG, quantitative electroencephalography.

EEG recordings have demonstrated the resurgence of synaptic function throughout the rewarming phase (Keenan et al. 2016). Rapid rewarming is associated with a disappearance of high‐frequency EEG activity indicating cerebral ischemia from a sudden rise in intracranial temperature. There is a systematic decline in the amplitude of aEEG as the core body temperature decreases, and electrical inactivity of aEEG indicates the least metabolic rate of the brain. Gradually symmetrical increase activities of aEEG during rewarming provide some assurance of postoperative neurologic function. Asymmetrical aEEG or abnormal QEEG may suggest inadequate brain protection or cerebral ischemia (Figure 10).

### 3.3 ICU Brain Function Monitoring Post TAAD Surgery

**FIGURE 11:**
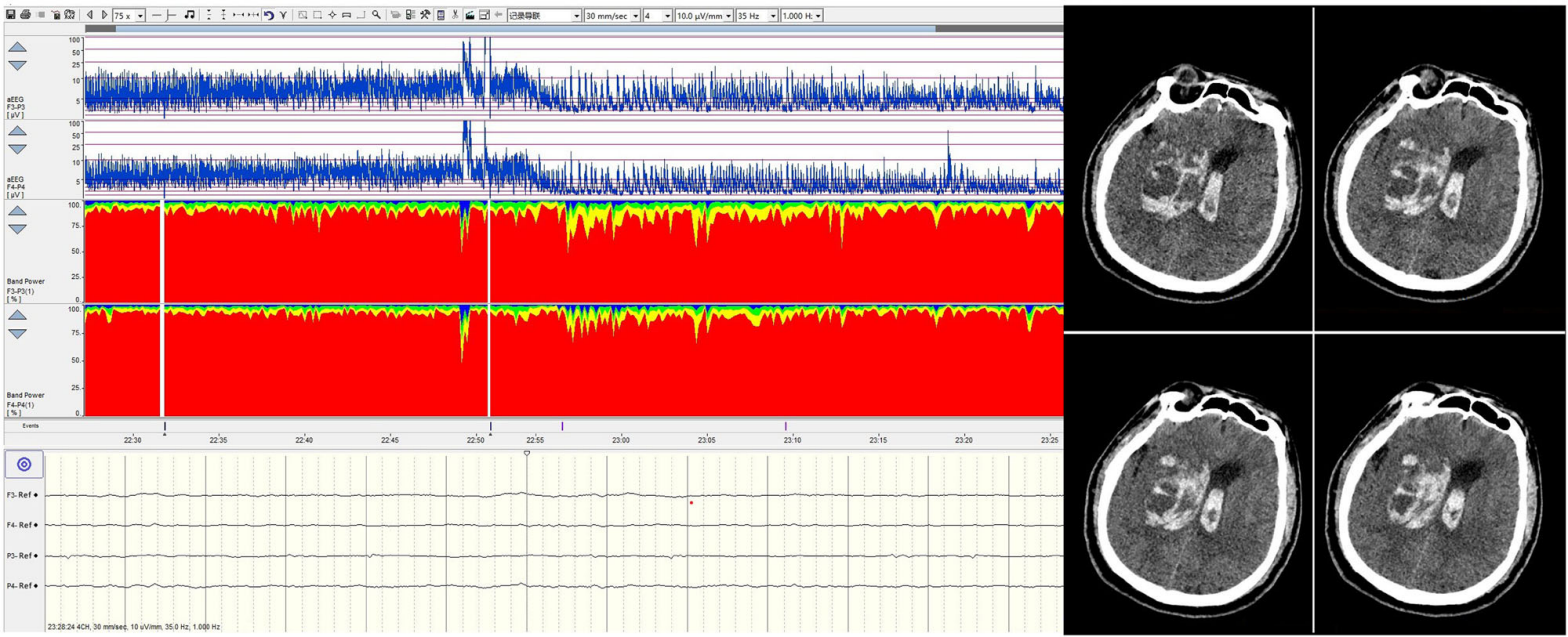
Post‐operative QEEG changes and correlation with cerebral hemorrhage on CT scan. post‐operation, 30 min of QEEG monitoring showed progressive aEEG suppression and the onset of atypical burst suppression patterns, followed by the detection of extensive cerebral hemorrhage on a CT scan within an hour. F3‐P3 and F4‐P4 corresponded to the left and right hemispheres. aEEG, amplitude‐integrated electroencephalography; QEEG, quantitative electroencephalography.

Return of aEEG to its baseline state is becoming one of the important determinants of the prognosis for nervous system functionality. Head CT, MRI, and cerebral angiography are the dominant diagnostic tools for ischemic or hemorrhagic stroke; however, MRI requires 2–6 h and CT up to 1–5 days to detect cerebral injuries. Furthermore, there are contraindications for CT and MRI. QEEG exhibited a high sensitivity to cortical ischemia. This sensitivity enabled the early identification of either focal or widespread brain dysfunctions at the condition's inception. QEEG monitoring emerged as a supplementary method to traditional neuroimaging, aiding in the prompt detection of reversible damage and informing timely clinical responses. In the case of one TAAD patient, QEEG monitoring conducted 1 h post‐surgery indicated significant impairment. An immediate cranial CT scan was performed, revealing extensive cerebral hemorrhage (Figure 11).

## 4 Discussion

This review highlights the clinical potential of QEEG in the perioperative management of TAAD patients, particularly emphasizing its advantages in continuous cerebral function monitoring. This potential is notably reflected in two key QEEG parameters: aEEG and RBP.

Research regarding the prognostic value of QEEG for brain function during the perioperative TAAD surgeries is relatively limited. Our retrospective study indicated that aEEG symmetry and the proportion of slow waves in QEEG have diagnostic and prognostic potential in assessing hemispheric ischemia in patients undergoing TAAD surgery, with the lower aEEG value being the strongest predictor for brain injury (Wang et al. 2023). The primary clinical application of aEEG lied in assessing hypoxic‐ischemic encephalopathy, encompassing cases of neonatal asphyxia and HIE resulting from cardiac arrest in adults, aiding in prognostic evaluations (Rundgren, Rosén, and Friberg 2006; Van Rooij et al. 2005). An abnormal aEEG pattern exhibited a sensitivity of 100% and specificity of 81.3% for severe hypoxic–ischemic encephalopathy, with a positive predictive value of 85.0% and a negative predictive value of 100% (Liu, Shao, and Wang 2007). Moreover, aEEG served to assess the extent of brain injury, subsequently evaluating prognosis, gauging treatment efficacy, and guiding clinical interventions (Abend et al. 2011). It also facilitated monitoring of seizures, including nonconvulsive status epilepticus, and offered supportive insights in determining brain death (Sutter, Semmlack, and Kaplan 2016). However, QEEG has limitations, particularly in its ability to detect deep cerebral injuries. The limited coverage of QEEG electrodes, especially when using 4‐lead configurations as in our cases, may not capture the activities of entire brain, potentially leading to underestimation and bias. QEEG is more intuitive than raw EEG; however, its interpretation still requires some knowledge of EEG, which can be challenging for clinicians with limited experience.

Although this review proposes the utility of QEEG, its clinical application and value on patient outcomes need to be further explored due to the limitation of sample size and single institution study. A large‐scale, multicentered, and randomized controlled trial is needed to corroborate our finding. Nevertheless, QEEG shows a potential to become one of the important modalities of perioperative cerebral monitoring in the management of patients of TAAD. Further research is necessary to validate these initial findings and determine the true clinical value of QEEG in this specific patient population.

Moreover, the manuscript currently falls short in providing a comprehensive comparison between QEEG and other established neuro‐monitoring techniques, such as TCD and NIRS. TCD measures blood flow in large intracranial vessels, such as the middle cerebral artery, and is a sensitive tool for detecting microemboli and macroemboli during cardiac surgery (Qu et al. 2021). When used at the appropriate angle, TCD can assess cerebral blood flow and its distribution, helping to optimize brain perfusion during antegrade circulatory arrest. In patients with incomplete Willis circles, TCD can prompt a shift from unilateral to bilateral cerebral perfusion (Ghazy et al. 2016). TCD has limitations, including operator dependence, limited image quality, potential signal loss due to skull thickness, and the restriction of measuring only blood flow direction and velocity.

NIRS measures cerebral oxygen saturation by analyzing the absorption characteristics of hemoglobin in the near‐infrared spectrum. The commonly used definitions of cerebral desaturation, on the basis of data from carotid endarterectomy, include a decrease of over 20% from baseline or a baseline value below 50% (Zheng et al. 2013). Although NIRS has shown some weak associations with postoperative stroke and cognitive dysfunction in small‐scale studies (Lewis et al. 2018), a systematic review of seven randomized controlled trials found no significant link between low NIRS values and stroke. Studies of aortic arch surgery cohorts indicated that NIRS detected interruptions in cerebral perfusion and shifts to bilateral antegrade perfusion but did not show a significant association with neurological dysfunction (Urbanski et al. 2013). NIRS also has limitations, primarily monitoring regions supplied by the anterior and middle cerebral arteries and distinguishing signals from only 3 to 4 cm depth. The extent of extracranial signal noise and variations in arterial and venous blood distribution may affect the accuracy of cerebral oxygen saturation measurements during different phases of aortic arch surgery.

Despite the strengths of each technique—such as QEEG's real‐time monitoring capabilities, NIRS's ease of use, and TCD's precise blood flow measurement—each has its limitations. QEEG may miss deep cerebral ischemia, NIRS may be affected by signal noise and blood volume assumptions, and TCD's effectiveness relies on operator skill and image quality. Given the lack of direct comparative studies, future research should focus on directly comparing the effectiveness, accuracy, and clinical application of different brain monitoring techniques. Multicenter and prospective studies could provide a more comprehensive assessment of the relative advantages and limitations of each technique, offering stronger evidence for clinical decision‐making. In conclusion, although QEEG is still in an exploratory phase for TAAD patients, it has shown efficacy in other domains, suggesting its potential in multimodal brain function monitoring. However, its broader application requires further research and technological advancements.

## Author Contributions

**Ya‐Peng Wang**: conceptualization, software, data curation, methodology, validation, investigation, writing–original draft, visualization. **Jason Z. Qu**: conceptualization, methodology, validation, formal analysis, supervision, writing–review and editing. **Dong‐Jin Wang**: conceptualization, supervision, resources, methodology, funding acquisition, writing–review and editing, visualization.

## Ethics Statement

The study and its protocol received approval from the Institutional Review Board of Nanjing Drum Tower Hospital (approval number: 2022‐034‐01). The trial adhered to the ethical guidelines outlined in the “Declaration of Helsinki,” the “Ethical Review Measures for Biomedical Research Involving Humans” issued by the National Health Commission of China, and other pertinent national statutes and regulations.

## Consent

Informed consent was obtained from either the study participants or their legally authorized representatives before their inclusion in the study.

## Conflicts of Interest

The authors declare no conflicts of interest.

### Peer Review

The peer review history for this article is available at https://publons.com/publon/10.1002/brb3.70086.

## Data Availability

Data will be made available on request.
